# *Plasmodium ovale sp.* and *Plasmodium malariae* in Africa: difficult items of business on the malaria eradication agenda

**DOI:** 10.1186/1475-2875-9-S2-I10

**Published:** 2010-10-20

**Authors:** Colin J Sutherland, Mary C Oguike, Valerie Smith, Martha Betson, Teun Bousema, Peter L Chiodini

**Affiliations:** 1 HPA Malaria Reference Laboratory, London School of Hygiene & Tropical Medicine (LSHTM), London WC1E7HT, UK; 2 Dept of Immunology & Infection, London School of Hygiene & Tropical Medicine (LSHTM), London WC1E 7HT, UK; 3 Dept of Clinical Parasitology, Hospital for Tropical Diseases, London WC1E 6JB, UK; 4 Dept of Zoology, Natural History Museum, London SW7 5BD, UK

## 

The elimination of malaria in Africa is sensibly focused on the challenge of *P. falciparum.* However the parasite species *P. ovale curtisi, P. ovale wallikeri* and *P. malariae* occur across the continent at population prevalences ranging from negligible, up to ~9% for *P. ovale sp.* and up to ~15% for *P. malariae.* All three species are able to recur in the successfully treated patient months or years after exposure, even if successful clearance of blood-stage infections has occurred through antimalarial therapy. We present some recent data from surveys in several African localities as well as analyses of Malaria Reference Laboratory data for patterns of presentation of these parasite species among imported malaria cases in the UK, and consider the possibility that a substantial hidden burden of infection exists. The public health implications and challenges posed by these malaria species to malaria eradication programmes will also be considered.

